# A triple-blinded crossover study to evaluate the short-term safety of sweet manioc starch for the treatment of glycogen storage disease type Ia

**DOI:** 10.1186/s13023-021-01877-3

**Published:** 2021-06-03

**Authors:** Vaneisse C. L. Monteiro, Bibiana M. de Oliveira, Bruna B. dos Santos, Fernanda Sperb-Ludwig, Lilia F. Refosco, Tatiele Nalin, Terry G. J. Derks, Carolina F. Moura de Souza, Ida V. D. Schwartz

**Affiliations:** 1 Post-Graduate Program in Genetics and Molecular Biology, Universidade Federal Do Rio Grande Do Sul, Ramiro Barcelos St., 2350, Porto Alegre, Brazil; 2 Basic Research and Advanced Investigations in Neurosciences Laboratory (B.R.A.I.N), Hospital de Clínicas de Porto Alegre, Ramiro Barcelos St., 2350, Porto Alegre, Brazil; 3 Ultragenyx Brasil Farmacêutica Ltda, Presidente Juscelino Kubitchek Avenue, São Paulo, SP 04543-011 Brazil; 4 Section of Metabolic Diseases, Beatrix Children’s Hospital, University Medical Center of Groningen, University of Groningen, PO Box 30001, 9700 RB Groningen, The Netherlands; 5 Medical Genetics Service, Hospital de Clínicas de Porto Alegre, Rua Ramiro Barcelos, 2350, Porto Alegre, RS 90035-003 Brazil; 6 Department of Genetics, Universidade Federal Do Rio Grande Do Sul, Porto Alegre, Brazil; 7 NUCLIMED, Center for Clinical Research, Hospital de Clínicas de Porto Alegre, Ramiro Barcelos St., 2350, Porto Alegre, Brazil

**Keywords:** Inborn errors of metabolism, Hepatic glycogen storage disease, Treatment strategies, Cornstarch, Sweet manioc starch, Dietary treatment

## Abstract

**Background:**

Glycogen storage disease type 1a (GSD Ia) is characterized by severe fasting hypoglycemia. The clinical management includes the administration of uncooked cornstarch (UCCS). Although such a diet approach is effective in achieving euglycemia, its impact on the quality of life of patients should be considered. In vitro analyses suggest a longer release of glucose when using sweet manioc starch (SMS).

**Methods:**

We compared the efficacy and safety of the administration of SMS and UCCS during a short-fasting challenge in patients with GSD Ia in a randomized, triple-blind, phase I/II, cross-over study. GSD Ia patients aged ≥ 16 years and treated with UCCS were enrolled. Participants were hospitalized for two consecutive nights, receiving UCCS or SMS in each night. After the administration of the starches, glucose, lactate and insulin levels were measured in 1-h interval throughout the hospitalization period. The procedures were interrupted after 10 h of fasting or in a hypoglycemic episode (< 3.88 mmol/L).

**Results:**

Eleven individuals (mean age: 21.6 ± 4.3 years; all presenting body mass index > 25 kg/m^2^) participated in the study. The average fasting period was 8.2 ± 2.0 h for SMS and 7.7 ± 2.3 h for UCCS (*p* = 0.04). SMS maintained euglycemia for a greater period over UCCS. Increased lactate concentrations were detected even in absence of hypoglycemia, not being influenced by the different starches investigated (*p* = 0.17). No significant difference was found in total cholesterol, HDL, triglycerides and uric acid levels in both arms. None of the patients showed severe adverse events.

**Conclusions:**

SMS appears to be non-inferior to UCCS in the maintenance of euglycemia, thus emerging as a promising alternative to the treatment of GSD Ia.

## Background

Glycogen Storage Diseases comprise distinct genetic disorders caused by alterations in the synthesis or degradation of glycogen [1]. Glycogen storage disease type 1a (GSD Ia), typically known as Von Gierke disease (OMIM #232200), is an autosomal recessive metabolic disorder caused by deficiency of the enzyme glucose-6-phosphatase (G6Pase) [2], encoded by the *G6PC* gene located in the chromosome 17q21.31 [3]. G6Pase is anchored in the endoplasmic reticulum lumen, being highly expressed in the liver, kidney and small intestine [4, 5]. The estimated prevalence of GSD Ia is about 1 in 100,000 live births [6].

GSD Ia results in dramatic metabolic alterations, especially in fasting periods. Due to the deficient endogenous glucose production, patients showed severe hypoglycemia, hypertriglyceridemia, hyperlipidemia and increased production of lactic and uric acids [6]. The clinical management is based on dietary treatment to maintain euglycemia (blood glucose > 4 mmol/L or 70 mg/dL) and prevent secondary metabolic disorders [7].

Dietary treatment strategies intend to provide a continuous source of glucose by nocturnal intragastric infusion of glucose or regular administration of uncooked cornstarch (UCCS) [8]. Other potential strategies involve medium-chain triacilglycerol supplementation [8] and gene therapy [9]. In this regard, UCCS is a polysaccharide with slow degradation and glucose release, therefore constituting an interesting option to maintain euglycemia. The recommended UCCS dosage depends on age, weight and period of the day. As reference the dosage consists of 1.6 to 2.5 g *per* kilogram of body weight every 3–4 h for younger children, and every 4–6 h for older children, adolescents, and adults [1, 8]. Although UCCS therapy has shown successful results, there is no optimal protocol that can broadly attend to all the treatment requirements for patients with GSD Ia. In addition, even though UCCS is supposed to be palatable, practical, to prevent excessive weight gain and maintain normal appetite with scarce adverse effects [10], the overtreatment can induce hyperinsulinemia and obesity [8].

Sweet manioc starch (SMS) is a culinary product extracted from cassava (*Manihot esculenta*). As this root is an important staple food crop in many developing (tropical, intertropical, and sub-Saharan) countries, starch is one of its major components (58.9% of the dry matter), being constituted by approximately 80% of amylopectin [11].

Nalin et al. [12, 13] evaluated the digestion of distinct starches brands from Brazil, United States of America and the Netherlands, including modified starch (Glycosade®, Vitaflo Ltda) and SMS (Fritz and Frida®) in a dynamic gastro-small intestine model (TIM-1). These authors showed that a slower glucose release was obtained from SMS compared to other starches. Moreover, their results also indicated that the digested amount of SMS was reduced compared to the other analyzed starches. Subsequently, the authors [12] have also evaluated the amylose/amylopectin ratio in same starch samples. Interestingly, SMS displayed slightly higher amounts of amylopectin.

Hypothetically, the slower glucose release induced by the digestion of SMS and its widespread availability at a relative low cost could constitute an interesting tool in the arsenal to prevent GSD Ia-induced hypoglycemia during fasting periods. In the light of the demand to develop new therapeutic technologies for GSD management [14], the present study aimed to assess the efficacy and safety of SMS administration in patients with GSD Ia.

## Results

Eleven GSD Ia participants (M: 6, F: 5) were enrolled in the study (mean age: 21.6 ± 4.3 years). All participants exhibited body mass index > 25 kg/m^2^ (mean: 28.2 ± 3.6 kg/m^2^). The clinical profile of the participants is summarized in Table 1. At baseline, four patients (A, C, G, and K) presented high lactate (> 2.2 mmol/L); six patients, high uric acid; and 10 patients, high triglycerides levels.

**Table 1:** Genetic and biochemical profile of the GSD Ia patients enrolled in study (n = 11, baseline)

ID	Genotype	Gender (M/F)	Age (years)	Comorbidities	Body weight (Kg)	Height (cm)	BMI (kg/m2)	Starch-dosage 4/4 h (cm)	Glucose (mmol/L)	Lactic acid (mmol/L)	Insulin (UI/mL)	Uric acid (mg/dL)	TC(mg/dL)	HDL (mg/dL)	TG (mg/dL)
A	c.[247C>T];[820G>A]	F	24	Adenomas, nephrolithiasis	72.1	160	**28.2**	45	5.3	**2.55**	**29.1**	**7.7**	**324**	**25**	**1997**
B	c.[113A>T];[1039C>T]	F	16	–	76.8	163	**28.9**	47	4.0	1.69	4.7	6.2	198	44	**307**
C	c. [247C>T];[247C>T]	F	27	Hepatomegaly, hepatic hypervascular nodule	64.2	149	**28.9**	60	5.6	**2.48**	8.9	6.8	**217**	36	**384**
D	c.[113A>T];[1039C>T]	F	16	–	71.9	166	**26.1**	61	6.1	1.74	**30.8**	**7.8**	156	**23**	**344**
E	c.[247C>T];[247 C>T]	M	23	Nephrolithiasis, hepatic steatosis	78.6	164	**29.2**	65	6.2	1.79	**34.0**	6.9	170	**22**	**383**
F	c.[247C>T];[809G>T]	F	21	Adenomas	59.0	153	**25.2**	68	5.3	1.38	**16.7**	6.5	144	35	122
G	c.[247C>T];[247C>T]	M	16	Nephrolithiasis	82.0	169	**28.7**	73	4.1	**3.10**	5.7	**8.2**	196	**21**	**484**
H	c.[189G>C];[1039C>T]	M	20	–	78.0	176	**25.2**	77	5.6	1.65	**22.3**	**8.5**	**246**	39	**746**
I	c.[1039C>T];[1039C>T]	M	22	–	109.5	170	**37.9**	78	5.9	1.93	**20.3**	**7.8**	**244**	**34**	**448**
J	c.[113A>T];[323C>T]	M	27	–	72.0	167	**25.8**	85	5.1	2.10	**14.9**	6.8	**338**	37	**608**
K	c.[247C>T];[563-3C>G]	M	26	Mild auditive loss	75.2	170	**26.0**	90	5.7	**3.51**	**43.2**	**10.0**	**242**	**30**	**553**

M: male, F: female. Reference values: glucose (> 3.8 mmol/L); lactate (0.5–2.2 mmol/L); insulin (1.4 -14 µUI/mL); total cholesterol (TC, < 200 mg/dL); HDL cholesterol (> 35 mg/dL); triglycerides (TG, < 150 mg/dL) and uric acid (3.4 -7 mg/dL). In bold, abnormal values

Participants consumed an average amount of UCCS of 408.6 ± 86.5 g/day or 0.9 ± 0.2 g/kg/dose before the study. For this trial, all participants were given 100 g (1.3 ± 0.2 g/kg/dose) of carbohydrate starch, either SMS or UCCS.

### Efficacy

**Fig. 1:**
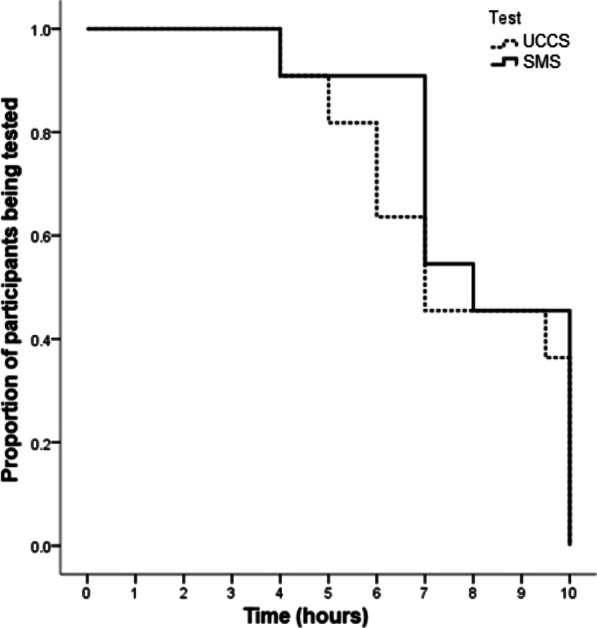
Kaplan Meier curve indicating test durations for each starch load performed (n = 11). UCCS: uncooked cornstarch and SMS: sweet manioc starch

Fasting time had a mean duration of 7.9 ± 1.8 h (SMS: 8.2 ± 2.0, UCCS: 7.7 ± 2.3, *p* = 0.04) (Fig. 1). The nadir time in euglycemia occurred in a 16-year-old male participant (Participant G), who remained in euglycemia during only 4 h after receiving any of the starches (SMS and UCCS). Four participants (B, D, E, I) remained in euglycemia during all the monitoring period (10 h) irrespective from the starch received. One participant fasted for 10 h after receiving SMS but not UCCS (Participant A). Under use of SMS, two patients (H and J) presented somnolence and fatigue, respectively, and had their tests interrupted at 7 h after the starch loading.

**Fig. 2:**
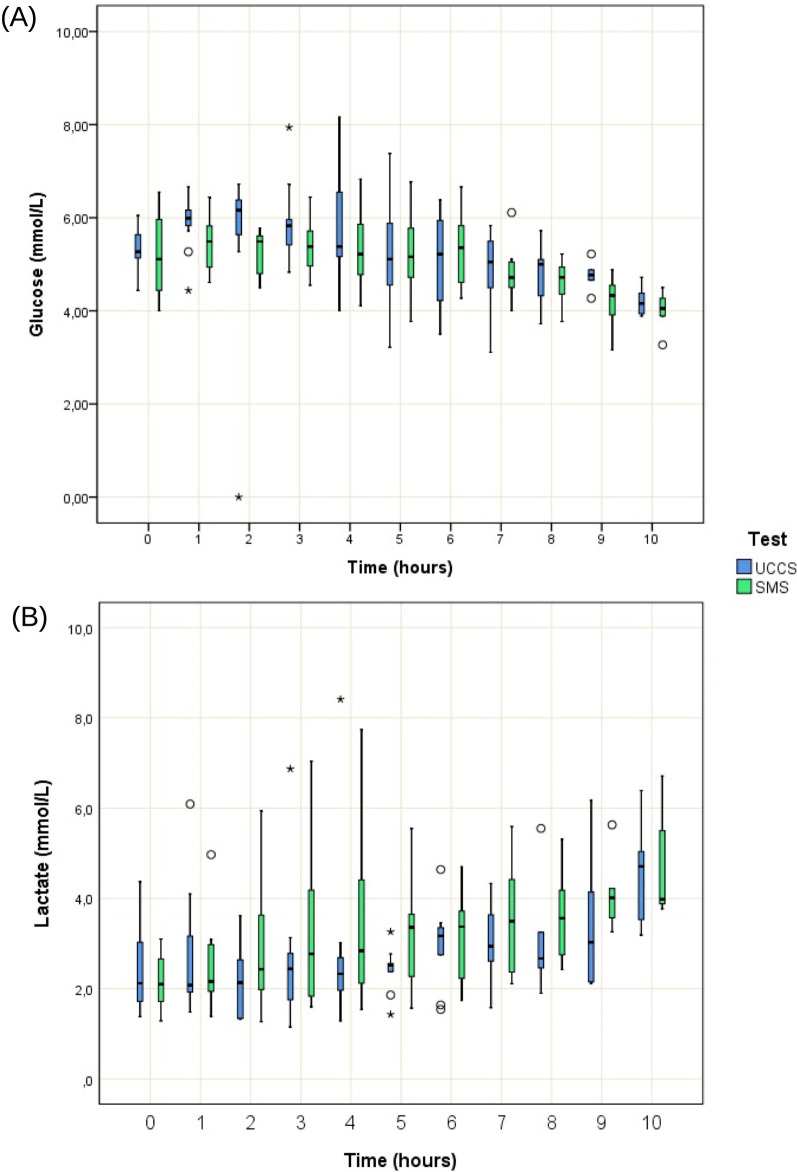
Blood concentrations of glucose and lactic acid after UCCS or SMS throughout the study period. **A** Glucose level for each starch load performed (n = 11), normal range: > 3.88 mmol/L; **B** Lactic acid levels for each starch load performed (n = 11), normal range: 0.5 to 2.2 mmol/L. UCCS (uncooked cornstarch load) or SMS (sweet manioc starch)

The SMS maintained euglycemia for a longer period (ANOVA, *p* = 0.04) and no carry-over effect was observed. In comparison to SMS, which had a more stable glycemic profile in the first 6 h after the load, the UCCS induced medians greater than 6.0 mmol/L in times T1 and T2, with the identification of glycemic peaks (Fig. 2A). All participants displayed similar lactate concentrations throughout the study evaluation (ANOVA, *p* = 0.17) and no carry-over effect was observed. An increase in lactate concentration was found even in the absence of hypoglycemia (Fig. 2B).

A carry-over effect was observed exclusively for the insulin levels regardless of the starch (*p* = 0.03).

Biochemical data of the participants (total cholesterol, HDL, triglycerides, and uric acid levels) are summarized in Table 2. No significant difference was found in these values when comparing both starches.

**Table 2:** Baseline and final levels of total cholesterol and fractions, triglycerides and uric acid(n = 11)

	Baseline (Mean ± SD)		Final (Mean ± SD)		Treatment effect (*p*)
	Before UCCS	Before SMS	After UCCS	After SMS	
TC (mg/dL)	221.7 ± 64.0	219.2 ± 50.0	234.9 ± 135.2	209.5 ± 55.8	0.77
TG (mg/dL)	589.8 ± 505.0	503.6 ± 243.6	456.6 ± 272.9	457.1 ± 216.3	0.14
HDL (mg/dL)	32.0 ± 8.6	31.2 ± 7.0	31.2 ± 9.1	31.1 ± 9.1	0.62
UA (mg/dL)	7.8 ± 1.0	7.3 ± 1.1	8.4 ± 1.3	8.1 ± 1.2	0.98

UCCS: uncooked cornstarch; SMS: sweet manioc starch; SD: standard deviation; TC: total cholesterol (< 200 mg/dL); TG: triglycerides (< 150 mg/dL); HDL: high density lipoprotein (> 35 mg/dL); UA: uric acid (3.4–7 mg/dL)

### Safety

None of the participants displayed serious adverse events. Mild hypoglycemia-related fatigue was reported in 3/11 participants when treated with UCCS and two under SMS. Two anxiety episodes were also reported in 2 participants treated with UCCS, which were clinically managed without need for medication. One participant displayed anxiety and tachycardia symptoms and stopped the daily protocol because of low levels of capillary blood glucose.

Eight participants (A, C, D, E, G, H, and K) presented high lactate levels (≥ 5 mmol/L) during the protocol. Among these patients, five presented lactate elevation exclusively under SMS, one exclusively under UCCS and two under both starches’ ingestion.

No gastrointestinal symptoms were reported and none of the participants discontinued the trial.

## Discussion

This randomized, triple-blinded pilot study revealed that SMS maintained blood glucose concentrations within the normal range for a longer period than the UCCS.

The advent of UCCS treatment brought many benefits to hepatic GSD patients. However, similar to all alternative dietary treatment for GSDs, adverse effects were also reported, including interrupted sleep for treatment, anxiety, exhaustion, risk of delayed administration [14] and food intolerance [15].

The negative impact of conservative treatment with UCCS has been causing concerns among health care professionals, patients and their families. This became clear with the publication of the consensus on research priorities for hepatic GSD, where one of the 11 cited items was “How can existing cornstarch preparations be modified or alternative treatments be implemented that are easier to administer and/or keep blood sugar levels more stable for patients with liver GSD?” [16]. To avoid these adverse effects, a modified experimental starch was proposed (the modified cornstarch, WMHM20) [10]. The authors concluded that the use of WMHM20 resulted in a longer duration of euglycemia and better short-term metabolic control. Subsequent studies proved its efficacy and safety [17, 18].

New products for assisting the nutritional management of hepatic GSDs have been consistently investigated. In 1986, Sidbury et al. [19] compared the effects of different raw starches, including arrowroot and tapioca, typical roots from South America. The authors have reported distinct patterns of starches absorption. In fact, both arrowroot and tapioca were less hydrolyzed than UCCS. However, UCCS was more efficient in maintaining euglycemia in patients with GSD Ia.

An in vitro study using a dynamic model of the gastrointestinal tract-1 (TIM-1) have demonstrated that the use of SMS resulted in a less rapidly available glucose in the glycemic index method and a higher resistant starch value. In addition, SMS led to a slower glucose release and minimal possible amount of indigestible material compared to UCCS. After 3 h of starches administration, only 55.5% the amount of SMS was digested while nearly 70% of UCCS was already digested [13]. The amylose/amylopectin ratio was also determined, reflecting the starch influence on the rate and the extent digestion. SMS presented a higher amount of amylopectin than UCCS, but not in sufficient amounts to fully explain the difference in digestibility [12].

In the present study, the SMS presented a more stable glycemic profile in the first 6 h and the intervention maintained glycemia within the recommended treatment interval described in guidelines. In patients with GSD, the amount and the quality of the ingested carbohydrates demand to be controlled in order to avoid hypoglycemia during fasting and increased levels of lactate, triglycerides and hepatomegaly. According to experts, the management of GSD should include small and frequent meals, favoring the complex carbohydrates over the simple carbohydrates [8]. This recommendation is based on the biochemical and nutritional properties of carbohydrates, which can critically determine the rate and extent of digestion and absorption in the small intestine. The greater is the release of glucose in the small intestine, the higher is the glucose blood bioavailability which favors the formation of glycogen. In general, granular starches with higher amylose content are more resistant to digestion, while greater amounts of amylopectin tend to be more easily digested. Other extrinsic aspects may also influence the starches digestion, such as its natural source, the granular structure, the degree of isolation as well as its processing and refinement [20].

Cassava starch consists of 80% amylopectin and 17–20% amylose [11]. Proportional amounts were found by Nalin et al. [11] in three Brazilian batch production. Additionally, SMS is constituted by approximately 170 g/kg of sucrose, trace amounts of fructose [10], with simple carbohydrates representing only 1 to 3% of the product [21]. Further analyses regarding the detailed composition of the studied starches are warranted.

The increased amylose/amylopectin ratio in SMS is associated with slow release of glucose and the maintenance of a prolonged euglycemia, thus constituting a promising alternative in the treatment of glycogen storage disorders, especially in Brazil, where access to slow-release starch is restricted to some patients. However, we also found an increased concentration of lactate irrespective from the starch, likely associated with the duration of fasting. Additional studies are necessary to identify possible starch components involved in hyperlactatemia.

The increased plasma lactate levels (> 2.2 mmol/L) verified in all patients even during euglycemia period deserves further investigation. As G6Pase also catalyzes one important step in gluconeogenesis [4] and this metabolic pathway is underactivated during euglycemia, the increased lactate levels may not be related to the gluconeogenesis. Hypothetically, such increased lactate concentrations could be directly associated with the metabolism of fructose or other sugars. The preparation of SMS using different cassava species, processing techniques of a mixture of brands could be employed in future trials. It also should be highlighted that four participants presented with high lactate levels and ten participants have displayed hypertriglyceridemia at the baseline evaluation, suggesting a previous poor metabolic control.

The main limitations of the present study are its short-term duration and that the evaluated dosages of the starches were distinct from that used in the pre-trial period.

## Conclusions

This study demonstrated a longer duration of euglycemia and greater stability of glucose levels in GSD Ia patients who underwent a short-term intervention with SMS, suggesting that this starch is a promising alternative in the treatment of this condition. Additional studies are warranted to understand the long-term effects of the administration of SMS and to identify possible starch components involved in hyperlactatemia.

## Methods

### Study design

This was a randomized, triple-blinded, phase I/II crossover study designed to evaluate the safety and efficacy of SMS in comparison to UCCS in preventing the hypoglycemia associated with GSD Ia. Participants were randomly assigned to groups receiving distinct starches. The principal investigators, the participants and the statistician were blind to the type of starch received. Only the researcher responsible for randomization and the study dietitian who dispensed study starches were not blinded to the type of starch administered. The study protocol included a hospitalization for two consecutive days and nights. At the time of hospital admission, anamnesis and physical examination (including weight and height assessment) were performed. All participants remained under their usual dietary treatment for GSD Ia during the day 1. The same dinner meal was served at 6 pm for all participants. At 10 pm, distinct randomized starches were orally administered. Patients remained with a permanent peripheral saline catheter, without any continuous infusion and without mobility restrictions.

Peripheral blood samples were collected at 10 pm (basal evaluation) to determine glucose, lactate, insulin, triglycerides, total cholesterol and HDL fraction and uric acid levels. Subsequently, the participants ingested 100 g of starch (UCCS or SMS) diluted in 200 mL of drinkable water. Blood samples were collected in a 1 h-interval following starch administration and the vital signs were checked. No additional food or beverages (water excepted) ingestion were allowed.

**Fig. 3:**
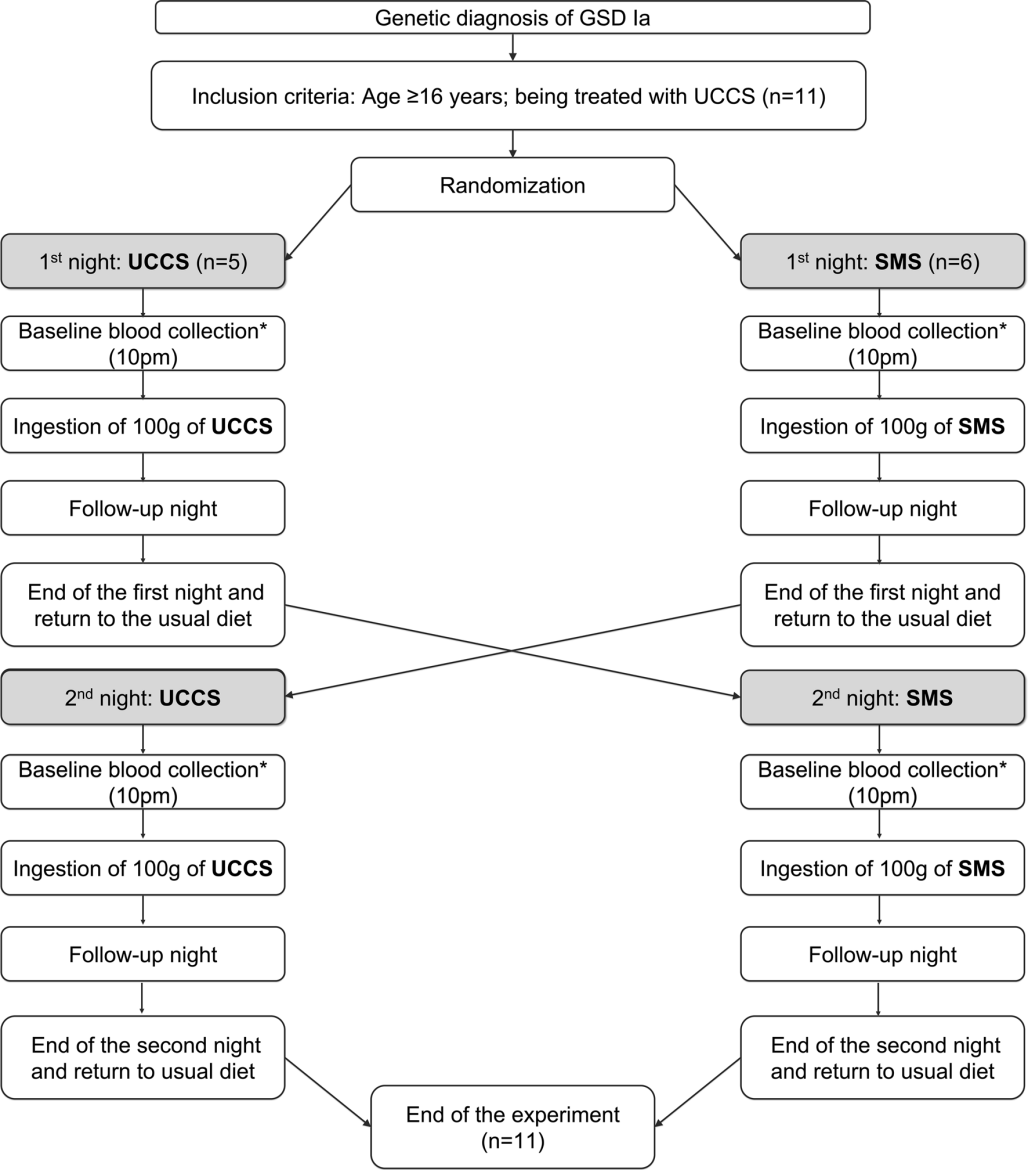
Study design. *refers to blood collection for the evaluation of glucose, lactic acid, insulin, total cholesterol, triglyceride and uric acid levels. **refers to blood collection for the glucose, lactic acid and insulin levels. GSD Ia: glycogen storage disorder type Ia; UCCS: uncooked cornstarch and SMS: sweet manioc starch

The fasting was interrupted at 8 am. The participants were allowed to follow their usual dietary treatment until 10 pm. After a standardized meal for dinner with an average of 50 g of carbohydrates, the participants received the switched starch, and the same evaluations were performed on the second night. The only modification was the type of starch administered (Fig. 3).

In case of hypoglycemia (blood glucose less than 3.88 mmol/L or 70 mg/dL) or symptoms of hypoglycemia, the fasting was discontinued immediately, and the participant received 10 g of glucose closely with the meal.

### Participants

To be eligible, participants should have clinical and genetic diagnosis of GSD Ia, be ≥ 16 years old, and be under UCCS therapy. All participants were seen at the Metabolic Disorders Clinics of Hospital das Clínicas in Porto Alegre, Brazil. Demographic data and clinical variables were retrieved from participants' medical records.

Participants received an anonymous reference number and were randomly assigned to receive SMS or UCCS in the first night of the study (Fig. 3). The starches were manufactured in accordance with the Brazilian standardized techniques for food quality and inspection. Physicians and dietitians planned a safe fasting for each participant before starting the trials.

### Tested products

Both starch samples were produced in Brazil (SMS from Fritz & Frida® and UCCS from Maizena®), similarly to the previous study of Nalin et al. [12]. All starch doses were administered as 100 g of raw powder diluted in 200 mL of water at room temperature as preconized for dietary treatment in GSD Ia. The high dose of starch (100 g) was determined in accordance with previous literature [18]. During the experimental procedures, both starches were stored in identical containers numbered in accordance with the randomization sequences by the study dietitian. The starches nutrition information is provided in Table 3.

**Table 3:** Starch nutrition facts

Nutrition Facts	UCCS	SMS
Portion (g)	20	20
Energy (kcal)	70	70
Protein (g)	0	0
Fat (g)	0	0
Carbohydrate (g)	17	17
Fiber (g)	0	0

*UCCS* uncooked cornstarch,* SMS* sweet manioc starch. Information provided by food brand owners in label data

### Randomization

Randomization was performed using an online software (www.randomization.com) by a researcher who was unaware of obtained clinical records. Blind data were maintained to all study personnel until the conclusion of statistical analysis, except for the study dietitian who prepared the starches doses for the participants. The researcher responsible for randomization and the study dietitian who dispensed study starches were not present in the tests and had no contact with the enrolled participants.

### Biochemical blood evaluation

Blood analysis was performed as follow: glucose (hexokinase colorimetric assay); lactate (colorimetric assay, normal range values (NRV): 0.5–2.2 mmol/L), insulin (chemiluminescent microparticle immunoassay, NRV: 1.4–14 µUI/mL), total cholesterol (enzymatic colorimetric assay, NRV: < 200 mg/dL), HDL cholesterol (homogeneous enzymatic colorimetric method, NRV: > 35 mg/dL), triglycerides (enzymatic colorimetric method, NRV: < 150 mg/dL) and uric acid (enzymatic colorimetric assay, NRV: 3.4–7 mg/dL). All analyses were performed by using a Cobas c702 analyzer and commercial kits. Insulin evaluation was performed using a Ci4100 analyzer. The plasma was frozen for 15 min after collection and then used in insulin evaluation.

### Study outcomes

The maintenance of euglycemia (blood glucose ≥ 3.88 mmol/L) was the primary endpoint of the study. The impact of dietary treatment on plasma lactate was considered a secondary endpoint.

### Statistical analysis

Categorical variables were represented as frequencies and percentages and continuous variables were presented as means and/or medians, standard deviation, and percentiles. The main data were analyzed as proposed by Altman (1991), which investigated period effects, treatment-by-period interactions, and treatment effects. The level of significance was established at 5% (*p* < 0.05). Analysis of variance (ANOVA) was used to compare the results and data were analyzed using the software SPSS v.18 and Stata V.

### Ethical aspects

The study was approved by the Ethics Board of the Hospital de Clínicas de Porto Alegre, Brazil (protocol #52645116500005327). The study design is registered in ClinicalTrials.gov (NCT03871673). All participants and their legal representatives read and signed the informed consent form before being enrolled in this study. This is an investigator-funded study.

## Data Availability

The datasets used and/or analysed during the current study are available from the corresponding author on reasonable request.

## Abbreviations

GSD Ia: Glycogen storage disease type 1a
UCCS: Uncooked cornstarch
SMS: Sweet manioc starch
G6Pase: Glucose-6-phosphatase enzyme
OMIM: Online Mendelian Inheritance in Man
TIM-1: Dynamic gastro-small intestine model
ANOVA: Analysis of variance
HDL: High density lipoprotein
WMHM20: Waxy Maize (Heat Modified) 20
NRV: Normal range values
